# Effectiveness of a calcium sodium phosphosilicate containing prophylaxis paste in reducing dentine hypersensitivity immediately and 4 weeks after a single application: a double-blind randomized controlled trial

**DOI:** 10.1111/jcpe.12057

**Published:** 2013-02-17

**Authors:** Klaus W Neuhaus, Jeffery L Milleman, Kimberly R Milleman, Kimberly A Mongiello, Thomas C Simonton, Courtney E Clark, Howard M Proskin, Rainer Seemann

**Affiliations:** 1 Department of Preventive, Restorative and Pediatric Dentistry, School of Dental Medicine, University of BernBern, Switzerland; 2 Salus Research, Inc.Fort Wayne, IN, USA; 3 DENTSPLY ProfessionalYork, PA, USA; 4 Howard M. Proskin & Associates, Inc.Rochester, NY, USA; 5 DENTSPLY DeTreyKonstanz, Germany

**Keywords:** calcium sodium phosphosilicate, clinical trial, dentine hypersensitivity, NovaMin®, prophylaxis paste

## Abstract

**Aims:**

The aim of this single-site, randomized, controlled, double-blind, 3-arm parallel study was to determine the effectiveness of a prophylaxis paste containing 15% calcium sodium phosphosilicate (CSPS; NovaMin®) with and without fluoride in reducing dentine hypersensitivity immediately after a single application and 28 days following dental scaling and root planing.

**Materials & Methods:**

Overall, 151 subjects were enrolled in this study. All subjects received a scaling and root planing procedure followed by a final prophylaxis step using one of three different prophylaxis pastes: Test-A (15% NovaMin® and NaF), Test-B (15% NovaMin®) and a control. Dentine hypersensitivity was assessed by tactile stimulus (Yeaple Probe®) and by air blast (Schiff scale) at baseline, immediately after and 28 days after a prophylaxis procedure. One hundred and forty-nine subjects completed the study.

**Results:**

Subjects having received the test prophylaxis pastes showed statistically lower (anova, *p* < 0.05) dentine hypersensitivity compared with the control group immediately after the prophylaxis procedure (Yeaple Probe®: Test-A = 20.9 ± 12.6, Test-B = 22.7 ± 12.9, Control=11.2 ± 3.1; Schiff score: Test-A = 1.1 ± 0.6, Test-B = 1.1 ± 0.6, Control = 2.0 ± 0.7) and after 28 days (Yeaple probe: Test-A = 21.5 ± 11.9, Test-B = 20.6 ± 11.3, Control = 11.8 ± 6.0; Schiff score: Test-A = 1.0 ± 0.6, Test-B = 1.0 ± 0.6, Control = 2.0 ± 0.7).

**Conclusions:**

In conclusion, the single application of both fluoridated and non-fluoridated prophylaxis pastes containing 15% CSPS (NovaMin®) provided a significant reduction of dentine hypersensitivity up to at least 28 days.

Dentine hypersensitivity is of common occurrence in the general adult population (Rees & Addy 2002), (Chabanski et al. 1996), (Dowell & Addy 1983) and has been defined as pain arising from exposed dentine in response to external thermal, tactile, osmotic or chemical stimuli, that cannot be explained by any other form of dental defect or pathology (Addy 1990).

The mechanism of tooth sensitivity can be explained by the widely accepted “hydrodynamic theory” developed by Brännström based on the initial observations of Gysi (Gysi 1900,Brannstrom & Astrom 1972). According to this theory, open tubules of exposed dentine allow the movement of dentinal fluid within the dentinal tubules indirectly stimulating the pulp nerves. Although the exact mechanism by which the fluid-flow stimulates mechanoreceptors within the dental pulp remains unknown (Chidchuangchai et al. 2007), histological studies reveal that compared to insensitive dentine, more widened dentine tubules and more open tubules per area can be found in sensitive dentine (Absi et al. 1987).

Therefore, chemical and/or mechanical occlusion of patent tubules has been reported as an effective method for tooth sensitivity reduction (Markowitz & Pashley 2007, 2008). A recent network meta-analysis confirmed that most active treatment options of dentine hypersensitivity (physical or chemical occlusion of open tubules, nerve desensitization, laser therapy or combinations thereof) act significantly better than placebo treatments (Lin et al. 2013).

Dental professionals may contribute to dentine exposure and dentine hypersensitivity by instrumentation. It is of common knowledge that patients often report increased hypersensitivity following scaling and root planing (von Troil et al. 2002). Thus, a prophylaxis paste with an immediate desensitizing effect used for stain removal and polishing after scaling and root planing procedures would be beneficial for the patient.

Recently, a new prophylaxis paste (Nupro® Sensodyne®, DENTSPLY Professional) has been brought to the market that contains 15% of a calcium sodium phosphosilicate (CSPS). CSPS is an inorganic amorphous material that was designed based on a class of materials known as bioactive glasses and marketed under the trade name NovaMin® (GlaxoSmithKline, London, UK). NovaMin® was originally developed as a bone regenerative material and recently has been engineered for oral care applications. The material has shown in vitro (Parkinson & Earl 2009) and in situ (West et al. 2011) to occlude dentine tubules, and is hypothesized to form a mechanically strong hydroxyapatite-like layer on the dentine surface, which can resist degradation by repeated acid challenges (Burwell et al. 2011, Earl et al. 2011). NovaMin® has shown to be effective in reducing dentine hypersensitivity when being used as an ingredient in toothpastes in concentration of 5% or 7.5% (Pradeep & Sharma 2010, Litkowski & Greenspan 2011, Salian et al. 2011, Sharma et al. 2011).

The primary purpose of this randomized controlled trial was to determine the effectiveness of a prophylaxis paste containing 15% calcium sodium phosphosilicate (NovaMin®), with and without fluoride, in reducing dentine hypersensitivity immediately after a single application following dental scaling and root planing. The secondary purpose was to assess the duration of sensitivity relief up to 28 days.

## Materials and Methods

### Overview and randomization

This clinical investigation was designed as a single-site, double-blind, randomized, three arm parallel group study involving subjects with hypersensitive teeth in accordance with the criteria described by Holland et al. (Holland et al. 1997). By means of screening and baseline exams, 151 subjects who demonstrated two hypersensitive teeth that satisfied the tactile and evaporative hypersensitivity enrolment criteria described below qualified to participate in the study. Qualified subjects were stratified based on their baseline tactile and evaporative hypersensitivity scores and were randomly defined within the strata to one of the three study treatments. All subjects received a scaling and root planing procedure followed by a final polishing step using one of three different prophylaxis pastes:

- Test-A: paste with 15% NovaMin®, with 2.7% sodium fluoride
- Test-B: paste with 15% NovaMin®, without fluoride
- Control: paste without NovaMin®, without fluoride

Table 1 summarizes the details of the prophylaxis pastes.

**Table 1:** Prophylaxis pastes product details

Group	Product	Ingredients
Test-A	NUPRO® Sensodyne® Prophylaxis Paste, with NovaMin® without fluoride (polish, spearmint) Lot # 11090809	Pumice and glycerin base, 15% NovaMin
Test-B	NUPRO® Sensodyne® Prophylaxis Paste, with NovaMin® with fluoride (polish, spearmint) Lot # 11090908	Pumice and glycerin base, 15% NovaMin 2.7% NaF
Control	NUPRO® Classic without fluoride (medium, mint) Lot # 10081802	Pumice and glycerin base

All subjects were given Crest regular toothpaste and an extra soft-bristled toothbrush to use twice daily (morning and evening) for 2 timed minutes during 14 days before the baseline examination and during the trial. No additional oral hygiene product or method was allowed other than the provided toothpaste and toothbrush. Tooth brushing was recorded in a treatment diary. Any new medication was recorded in a medication log.

### Sample size calculation

**Figure 1:**
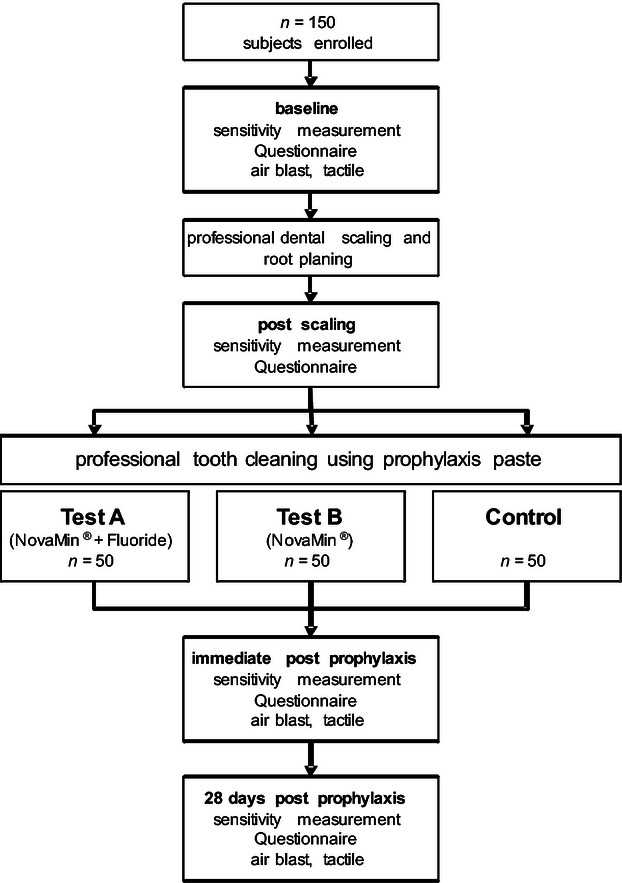
Summary flow-chart of study design

Based on the findings from a previous unpublished study, a sample size of 45 subjects per treatment group was found to be adequate to ensure 0.8 or higher power to detect a statistically significant difference in mean air blast sensitivity scores between a pair of treatments should the actual difference between those treatments be 0.90 or greater (two-sided comparison, *α* = 0.05). To account for the possibility of up to 10% dropouts (up to 15 subjects), 50 subjects per treatment group were aimed to be recruited (Fig. 1).

### Randomization

Excel software (Microsoft, Redmond, VA, USA) was used for randomization. The function = RAND() was used in column A for 300 random numbers. In column B, the letters A, B and C were put in groups of 3, 100 times (i.e. A, B, C, A, B, C).

The cells were then blocked in groups of 3 and randomized by the random number in column A from smallest number to the largest number. Doing it in blocks of 3 ensured that the groups were evenly distributed.

The mechanism used to implement the random allocation sequence was a randomization sheet. From the original excel sheet of numbers, 100 randomized letters were used for the patients who had a Schiff scale of 1, the next 100 randomized letters were used for the patients who had a Schiff scale of 2 and the last 100 randomized letters were used for the patients who had a Schiff scale of 3. As the patients were accepted into the study, they were marked (by a patient ID #) on the applicable randomization sheet (based on their Schiff score) and were assigned to the next letter (group) on the sheet.

The random allocation sequence was generated by K.A.M. and given to Salus Research before the start of the study. Salus Research enrolled the patients and the patients were assigned to the specific group numbers.

### Blinding

The entire study was blinded. The prophylaxis paste cups used had silver/blank lidstock and were only identified by a letter on the lidstock. The groups were not known by the examiners or patients. The examiner was in a different section of the building and the study coordinator gave the paste to the hygienist in yet another location of the building.

### Study population

Approval to conduct this clinical study using human subjects for research was granted by the U.S. Investigational Review Board (USIRB) according to the criteria of Helsinki. After IRB approval (U.S.IRB2011SRI/03), 327 persons were queried from the Salus Research categorized database as subjects with sensitive teeth and were contacted about this study. A total of 199 of them signed an informed consent form and were screened, with 161 accepted then appointed for the baseline visit 2 weeks later. At the baseline exam, 159 subjects were evaluated and 151 were randomized and enrolled into the study. There were 10 subjects who were lost following the screening exams and at the baseline assessment, 5 of which were disqualified due to insufficient tactile response with the Yeaple probe, 3 were disqualified due to a lack of evaporative hypersensitivity and the other 2 had scheduling conflicts and could not make their appointments. At the Day 28 examination, 149 evaluable subjects completed the study. A total of 2 subjects were lost following the baseline visit, both due to scheduling conflicts.

### Inclusion and exclusion criteria

The inclusion criteria for participating in the study were as follows: male and female subjects, 18–70 years of age with a minimum of two hypersensitive teeth not adjacent to each other; qualifying response to a tactile stimulus ≤20 grams and qualifying response to an air blast stimulus as defined by a score of ≥1 on the Schiff Cold Air Sensitivity Scale; good general health with no known allergies to the products being tested; use of a non-desensitizing dentifrice 2 weeks prior to the study and a minimum of 10 evaluable natural teeth excluding third molars.

Participants were excluded when one of the following criteria was met: oral pathology, causing pain similar to tooth sensitivity; chronic disease or allergy to test products, oral care products, personal consumer care products or their ingredients; pregnancy or lactation; requirement of anaesthesia during scaling; large amounts of calculus; infectious diseases such as HIV or tuberculosis; any condition requiring antibiotic prophylaxis for dental treatment; prophylaxis procedure within 30 days prior to the screening appointment; desensitizing or bleaching treatment within 90 days prior to the screening appointment; excessive gingivitis, advanced periodontal disease or treatment for periodontal disease within 12 month prior to the screening appointment; sensitive teeth with mobility greater than score 1; regular use of sedatives, anti-inflammatory drugs or analgesics; participation in a desensitizing dentifrice study within the past 4 weeks; current participation in any other clinical study or receipt of an investigational drug within 30 days prior to the screening visit; being an employer of the sponsor or member or relative of the study site staff directly involved in the study.

### Treatment procedure

There were no changes to trial outcomes after the trial commenced. The scaling and root planing procedure was carried out by licensed dental professionals using standard hand instruments. As the last step of this prophylaxis procedure, the assigned prophylaxis paste was applied for one minute as described in the instructions for use by means of a soft cup disposable contra angle hand piece (DENTSPLY Professional, York, PA, USA).

### Hypersensitivity assessment

All hypersensitivity measurements were performed by one examiner using the following three methods at the time-points described in Fig. 1. Tactile hypersensitivity was assessed by using a calibrated Yeaple Probe (electronic force-sensing probe model 200A, XiniX Research, Portsmouth, NH, USA) as described elsewhere (Gillam et al. 1992). The accuracy of the probe was checked daily. The probe tip was placed perpendicular to the evaluable tooth surfaces, just apical to the cemento enamel junction and drawn slowly across the surface in a distal to mesial direction to ensure application of the stimulus across all patent tubules. After each challenge, subjects were asked to indicate whether the sensation was painful. Only “Yes” or “No” answers were accepted. Testing began at 10 grams and increased by 10 grams with each successive challenge until a “Yes” response has been challenged or 50 grams were reached. The grams setting, which elicited the “Yes” response, was then repeated. If a second “Yes” response was not obtained the gram setting was increased until two consecutive “Yes” responses could be found. The respective gram setting was recorded as threshold value. The upper test value was 50 grams. If no sensitivity was found, the threshold was recorded as >50 grams.

Air blast hypersensitivity was assessed by applying a one-second blast of air from a calibrated (air pressure of 60 PSI/0.414 N*mm^−2^, checked daily for accuracy) standard dental unit air syringe directed to the exposed buccal site of the hypersensitive tooth using a 4-point scale: 0 = no response; 1 = response + subject does not request discontinuation of stimulus; 2 = response + subject requests discontinuation of stimulus; 3 = pain + subject requests discontinuation of stimulus (Schiff et al. 1994).

#### Questionnaire

All subjects completed a questionnaire titled to assess their whole-mouth, tooth sensitivity at selected time-points as follows: prior to the baseline assessments, immediately following scaling and root planing, immediately following the timed, 1-minute prophylaxis paste application, and prior to the 28-day assessments. The questionnaire contained a 4-item verbal descriptor scale: Score 1: no discomfort or awareness of sensitivity; Score 2: mild discomfort/pain from sensitive teeth; Score 3: moderate discomfort/pain from sensitive teeth; Score 4: severe pain from sensitive teeth.

### Statistical methods

Within treatment comparisons of the baseline versus the follow-up values were performed using paired *t*-tests. Comparisons between treatment groups at post-baseline time-points were performed using analyses of covariance (ancova), in which the baseline scores were employed as a co-variable. All comparative statistical tests were two-sided, and employed a level of significance of 0.05. All analyses were performed using SAS, release 9.3 (SAS Institute Inc., Cary, NC, USA).

## Results

### Demographic characteristics

The 151 subjects enrolled at the baseline assessments study were comprised of 116 females and 35 males. A total of 149 subjects completed the entire study with a gender distribution of 115 females (average age 43 years) and 34 males (average age 39 years). The trial ended when the selected patients were treated.

### Hypersensitivity

#### Yeaple Probe®

The Yeaple Probe® hypersensitivity data are summarized in Table 2a. All groups (*n* = 151) were evenly balanced with no statistically significant differences for the pre-prophylaxis baseline values. Mean values ranged from 10.60 (Control) to 10.38 grams (Test-B). Immediately following the scaling/root planing and prophylaxis procedure, the mean values resulted in statistically significant (*p* < 0.0001) improvements for both test groups compared with the control. There was no statistical difference between the test groups. The mean relative improvement from baseline immediately after the prophylaxis procedure was 100.5% for Test-A and 119.1% for Test-B.

**Table 2a:** Tactile sensitivity scores; between-treatment *p*-values for the baseline visit are calculated using an anova model that includes fixed factor treatment

Assessment/Treatment		Tactile sensitivity						Change from baseline							Between-Treatment *p*-value		
				
		*n*	Mean	SD	Median	Min.	Max.	*n*	Mean	SD	Median	Min.	Max.	*p*-val	vs. Test-A	vs. Test-B	vs. Control
Baseline	Test-A	49	10.41	1.38	10.00	10.0	15.0	0	–	–	–	–	–	–	–	n.s.	n.s.
	Test-B	52	10.38	1.35	10.00	10.0	15.0	0	–	–	–	–	–	–	n.s.	–	n.s.
	Control	50	10.60	1.64	10.00	10.0	15.0	0	–	–	–	–	–	–	n.s.	n.s.	–
Post-Prophy	Test-A	49	20.87	12.64	15.00	10.0	55.0	49	10.46	12.58	5.00	0.0	45.0	<0.0001	–	n.s.	<.0001
	Test-B	52	22.74	12.99	20.00	10.0	55.0	52	12.36	12.91	10.00	−5.0	45.0	<0.0001	n.s.	–	<.0001
	Control	50	11.20	3.12	10.00	10.0	25.0	50	0.60	3.45	0.00	−5.0	15.0	n.s.	<0.0001	<.0001	–
Day 28	Test-A	49	21.48	11.86	20.00	10.0	55.0	49	11.07	11.90	10.00	0.0	45.0	<0.0001	–	n.s.	<.0001
	Test-B	52	20.58	11.32	17.50	10.0	55.0	52	10.19	11.37	5.00	−5.0	45.0	<0.0001	n.s.	–	<.0001
	Control	48	11.77	5.95	10.00	10.0	37.5	48	1.15	5.88	0.00	−5.0	27.5	n.s.	<0.0001	<.0001	–

Between-treatment *p*-values for the subsequent visits are calculated using an ancova model that includes fixed factor treatment, and the baseline tactile sensitivity score as a covariate.

n.s. = non-significant.

The Day 28 Yeaple Probe® exams resulted in statistically significant (*p* < 0.0001) mean value differences for the test groups (21.48 and 20.58 grams) compared to the control (11.77 grams). Moreover, the tactile sensitivity mean value relationships achieved with the single polish application at the baseline appointment were nearly identical at day 28 for all groups and the statistical significant relationships were maintained (*p* < 0.0001). The mean relative improvement from baseline 28 days after the prophylaxis procedure was 106.3% for Test-A and 98.3% for Test-B (Fig. S1).

Cross-tabulations show an improvement of tactile sensitivity in about half of the test sites both post-prophy and after 28 days, but almost no change in the control sites (Table 2b).

**Table 2b:** Summary of tooth-wise tactile sensitivity scores. Number of subjects exhibiting each non-missing score; transition baseline to Post-Prophy and Day 28

Baseline score		Post-Prophy score						Day 28 score					
			
		10	20	30	40	50	> 55	10	20	30	40	50	> 55
Test-A	10	53	14	9	8	3	7	44	18	19	5	1	7
	20		2	1	1			1	1	1	1		
	30												
	40												
	50												
	> 55												
Test-B	10	43	22	15	9	4	7	49	22	16	6	1	6
	20	1		3				1	1	1		1	
	30												
	40												
	50												
	> 55												
Control	10	86	6	2				83	4		1		2
	20	4	2					5	1				
	30												
	40												
	50												
	> 55												

Within each treatment, cells above the diagonal represent sensitivity reductions between the time-points.

#### Air Blast (Schiff Scale)

The three groups were evenly balanced with no statistically significant differences for the baseline values (Table 3a). The mean values (4-point scale) ranged from 1.97 in the control to 1.93 in both test groups. Immediately following the scaling/root planing and prophylaxis procedure, the Schiff scale sensitivity values for the test groups were statistically significantly (*p* < 0.0001) less sensitive than the control (Table 3a). The mean relative improvement from baseline immediately after the prophylaxis procedure was 44.6% for both Test-A and Test-B (Fig. S2).

**Table 3a:** Between-treatment *p*-values for the baseline visit are calculated using an anova model that includes fixed factor treatment

Assessment/Treatment		Air blast sensitivity						Change from baseline							Between-Treatment *p*-value		
			
		*n*	Mean	SD	Median	Min.	Max.	*n*	Mean	SD	Median	Min.	Max.	*p*-val	vs. Test-A	vs. Test-B	vs. Control
Baseline	Test-A	49	1.93	0.59	2.00	1.0	3.0	0	–	–	–	–	–	–	–	n.s.	n.s.
	Test-B	52	1.93	0.59	2.00	1.0	3.0	0	–	–	–	–	–	–	n.s.	–	n.s.
	Control	50	1.97	0.63	2.00	1.0	3.0	0	–	–	–	–	–	–	n.s.	n.s.	–
Post-Prophy	Test-A	49	1.07	0.64	1.00	0.0	3.0	49	−0.86	0.62	−1.00	−2.5	0.5	<0.0001	–	n.s.	<0.0001
	Test-B	52	1.07	0.62	1.00	0.0	2.5	52	−0.87	0.71	−1.00	−2.5	0.0	<0.0001	n.s.	–	<0.0001
	Control	50	1.97	0.70	2.00	0.0	3.0	50	0.00	0.35	0.00	−1.0	1.0	n.s.	<0.0001	<0.0001	–
Day 28	Test-A	49	0.98	0.62	1.00	0.0	3.0	49	−0.95	0.66	−1.00	−2.5	0.5	<0.0001	–	n.s.	<0.0001
	Test-B	52	0.99	0.55	1.00	0.0	2.5	52	−0.94	0.68	−1.00	−2.5	0.5	<0.0001	n.s.	–	<0.0001
	Control	48	2.03	0.67	2.00	0.5	3.0	48	0.03	0.47	0.00	−1.0	1.0	n.s.	<0.0001	<0.0001	–

Between-treatment *p*-values for the subsequent visits are calculated using an ancova model that includes fixed factor treatment, and the baseline air blast sensitivity score as a covariate.

n.s. = non-significant.

The Day 28 assessments resulted in statistically significant (*p* < 0.0001) lower values for the test groups (0.98 and 0.99) compared to the control (2.03). In addition, the within-group relationships resulting from the single prophylaxis paste application at the baseline prophylaxis were nearly identical at the 4-week exam and the between group statistically significant reductions in air blast sensitivity were maintained (*p* < 0.0001) for the entire study. The mean relative improvement from baseline 28 days after the prophylaxis procedure was 49.2% for Test-A and 48.7% for Test-B.

Cross-tabulations show an improvement of air blast sensitivity in about 70% both post-prophy and after 28 days in both test groups, whereas the control group remained largely unchanged (Table 3b).

**Table 3b:** Summary of tooth-wise Schiff Air Blast Sensitivity Scores. Number of subjects exhibiting each non-missing score; transition. Baseline to Post-Prophy and Day 28

Baseline score		Post-Prophy score				Day 28 score			
		
		0	1	2	3	0	1	2	3
Test-A	0								
	1	9	16	2		10	16	1	
	2	12	28	11		12	32	7	
	3	2	4	11	3	2	7	8	3
Test-B	0								
	1	9	19			11	15	2	
	2	10	27	18		12	27	16	
	3	2	11	6	2	4	10	6	1
Control	0								
	1	5	13	8		2	15	6	
	2	2	1	43	5	1	4	33	12
	3			3	20			7	16

Within each treatment, cells below the diagonal represent sensitivity reductions between the time-points.

### Sensitivity questionnaire

The baseline summary of the questionnaire scores demonstrated that the Test group A and the control were nearly identical in relation to their frequency distributions among the 4 levels of self-reported sensitivity (Table 4). In contrast, Test group B revealed fewer subjects with moderate sensitivity and more subjects with no discomfort or awareness of sensitivity.

**Table 4:** Summary of self-assessed sensitivity (questionnaire); between-treatment *p*-values for the baseline visit are calculated using an anova model that includes fixed factor treatment

Assessment/Treatment		Subject-Assessed sensitivity						Change from baseline							Between-Treatment *p*-value		
				
		n	Mean	S.D.	Median	Min.	Max.	n	Mean	S.D.	Median	Min.	Max.	*p*-values	vs. Test-A	vs. Test-B	vs. Control
Baseline	Test-A	49	0.90	0.82	1	0	3	0							–	n.s.	n.s.
	Test-B	52	0.67	0.68	1	0	2	0							n.s.	–	n.s.
	Control	50	0.86	0.76	1	0	2	0							n.s.	n.s.	–
Post-Scaling/Root Planing	Test-A	49	1.31	0.85	1	0	3	49	0.41	0.93	0	−2	3	0.0036	–	n.s.	n.s.
	Test-B	52	1.19	0.72	1	0	3	52	0.52	0.67	1	−1	2	0.0002	n.s.	–	n.s.
	Control	50	1.36	0.75	1	0	3	50	0.50	0.89	0	−2	3	<0.0001	n.s.	n.s.	–
Post-Prophy	Test-A	49	1.14	0.76	1	0	3	49	0.24	0.90	0	−2	3	n.s.	–	n.s.	n.s.
	Test-B	52	0.96	0.74	1	0	2	52	0.29	0.78	0	−2	2	0.0203	n.s.	–	n.s.
	Control	50	1.20	0.90	1	0	3	50	0.34	1.00	0	−2	2	0.0098	n.s.	n.s.	–
Day 28	Test-A	49	0.47	0.62	0	0	3	49	−0.43	0.82	0	−3	1	0.0006	–	n.s.	0.0010
	Test-B	52	0.52	0.64	0	0	2	52	−0.15	0.80	0	−2	2	n.s.	n.s.	–	0.0236
	Control	48	0.88	0.79	1	0	3	48	0.02	0.70	0	−2	3	n.s.	0.0010	0.0236	–

Between-treatment *p*-values for the subsequent visits are calculated using an ancova model that includes fixed factor treatment, and the baseline Subject-Assessed sensitivity score as a covariate.

n.s. = non-significant.

Immediately following the scaling and root planing procedures and following the prophylaxis procedure, all three groups showed an increase in sensitivity awareness. The statistical comparison demonstrated that none of the treatment groups were different from one another.

At Day 28, the categorical summary of questionnaire scores revealed a large shift to the “no discomfort or awareness of sensitivity” for the test groups compared to the control. Interestingly, approximately 57% of subjects in the test groups self-reported “no discomfort” at Day 28 compared to their Baseline values of 37% (Test-A) and 44% (Test-B). In comparison, the control group demonstrated a decrease in the self-reported “no discomfort” category from 36% (Baseline) to 32% (Day 28) (data not shown in Table). The statistical analysis resulted in statistically significant reductions for both test groups compared to the control. No adverse effects on the oral mucosa were reported during the trial.

## Discussion

This study showed that the single professional application of a prophylaxis paste containing CSPS was able to significantly reduce dentinal hypersensitivity immediately and 28 days after scaling and root planing procedures. This effect was independent from the presence of fluoride in the prophylaxis paste.

To minimize dentinal hypersensitivity, mechanical occlusion of open dentinal tubules and/or down-regulation of nerval response have been described as effective means (Wara-aswapati et al. 2005). The mode of action of CSPS has been investigated in vitro and was described to consist in the formation of a chemically and mechanically stable apatite-like calcium phosphate hydroxycarbonate layer (Litkowski et al. 1997, Earl et al. 2011). The initial reactivity of CSPS particles was found to form a negative charge on the surface of exposed dentine in vitro, enabling establishment of covalent bindings of CSPS to side groups of Type 1 dentinal collagen fibres (Orefice et al. 2009). Local precipitation of apatite-like material was attributed to immediate release of sodium ions when CSPS comes in contact with water or saliva. This induces a rise of the local environmental pH, which subsequently facilitates release of calcium and phosphate ions (Andersson & Kangasniemi 1991).

In our study, the desensitizing effect immediately after application of the prophylaxis paste could be detected by air blast and tactile stimulus, but not by whole-mouth tooth sensitivity questionnaire (Table 4). This was expected because the questionnaire was designed to assess hypersensitivity experience in every-day challenges to the teeth. Twenty-eight days after the prophylaxis procedure, the self-reported subjective pain was significantly lower compared with baseline (Table 4) supporting the reduced level of hypersensitivity also found by air blast and tactile stimulus after 28 days (Tables 2 and 3). In future clinical studies on dentine hypersensitivity, functional magnetic resonance imaging (fMRI) could be additionally applied as a method to objectively assess pain (Meier et al. 2012).

Brushing habits including the brushing frequency were reported to significantly correlate with hypersensitivity (Gillam et al. 1999, West 2006). To meet this possible confounder, oral self-care was standardized as each participant was provided a conventional non-desensitizing fluoridated toothpaste and an adult extra soft-bristled toothbrush, and was instructed to brush twice daily using only the provided material. Other habits and possible confounders such as drinking acidic beverages were not assessed in this study. These factors based on personal preferences are difficult to assess and difficult if not practically impossible to standardize. However, the study population was well-balanced in terms of measurable factors such as age and distribution of sensitivity scores at baseline, proven by low standard deviations in all groups. The proportion of females included in our study was larger than males but reflected the pool of patients that were originally asked to participate. A recent review stated that dentine hypersensitivity appears to be more frequent in females (West 2008), although the biological stimulus is the same in both genders. Whether increased hypersensitivity is attributable to different pain perception or different awareness of pain is matter of speculation. However, most probably it cannot be regarded as a confounding factor in this trial.

Patient compliance with supportive periodontal therapy is generally low (30% or less; (Kerry 1995)), and pain or fear of pain contributes to this picture (Hoffmann et al. 2005). Dentine hypersensitivity is commonly observed and reported after scaling and root planing. Adding desensitizing agents into a prophylaxis paste thus aims at increasing patient comfort immediately after periodontal therapy and thus might contribute to better compliance. In contrast to desensitizing daily toothpaste products, where a pain relief is expected over time, the single application of a desensitizing prophylaxis paste results in immediate pain relief, which was shown in our study. Another desensitizing paste containing 8% arginine and calcium carbonate was shown to lead to significant reduction of dentine hypersensitivity when applied as single professional application prior to (Hamlin et al. 2009) or during professional tooth cleaning (Schiff et al. 2009). Other than the latter study using the same prophylaxis paste as in our study as control (Nupro)(Hamlin et al. 2009, Schiff et al. 2009) this study included a true matched control by comparing identical polishing pastes with and without the active agent NovaMin®.

A clinical recommendation whether CSPS prophylaxis pastes or arginine/calcium carbonate containing desensitizing pastes are more suitable for dentine hypersensitivity treatment in conjunction with prophylaxis procedures is currently not possible based on the available literature.

## Conclusions

The single application of both fluoridated and non-fluoridated prophylaxis pastes containing 15% CSPS (NovaMin®) provided a significant immediate reduction of dentine hypersensitivity up to at least 28 days.

## Authorship disclosure

Conception and design of the study: KAM, TCS, CEC, RS, Acquisition of data: JLM, KRM, Analysis and interpretation of data: KWN, RS, HMP, Drafting the article: KWN, RS, Revising the article critically for important intellectual content: KAM, TCS, CEC, JLM, KRM, HMP, Final approval of the version to be published: all authors.

## Clinical Relevance

*Scientific rationale for the study*: Calcium sodium phosphosilicate (CSPS) acts through formation of a chemically and mechanically stable layer on the tooth surface. It binds to dentinal collagen fibres and promotes local precipitation of apatite-like material.

*Principal findings*: The single application of both fluoridated and non-fluoridated prophylaxis pastes containing 15% CSPS (NovaMin®) provided a significant reduction of dentine hypersensitivity up to at least 28 days.

*Practical implications*: Dental professionals may contribute to dentine exposure and dentine hypersensitivity by instrumentation. Thus, a prophylaxis paste with an immediate desensitizing effect used for stain removal and polishing after scaling and root planing procedures would be beneficial for the patient.

## Supporting Information

**Figure S1.** Relative tactile sensitivity improvement from baseline, **p*<0.001.

**Figure S2.** Relative air blast sensitivity improvement from baseline, **p*<0.001.
