# Supplementary figures and images for: Effectiveness of a calcium sodium phosphosilicate containing prophylaxis paste in reducing dentine hypersensitivity immediately and 4 weeks after a single application: a double-blind randomized controlled trial
Source: J Clin Periodontol. 2013 Feb 17;40(4):349–57. doi: 10.1111/jcpe.12057 (PMC3675645; doi:10.1111/jcpe.12057)

**Figure S1**


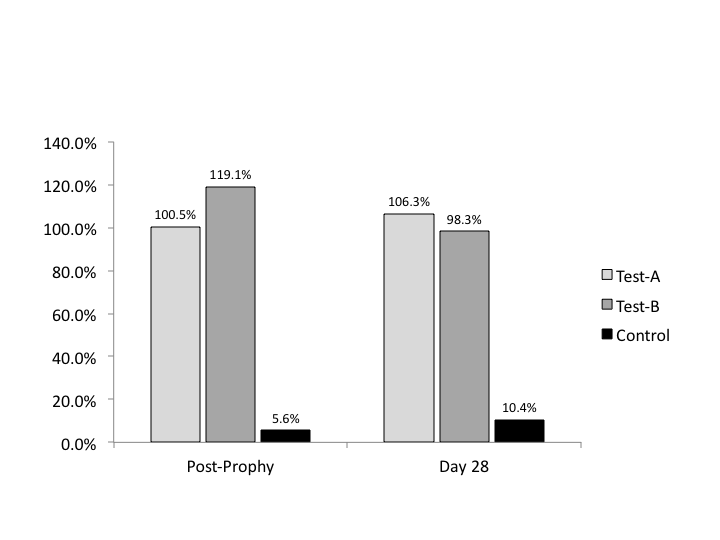

Supplement: Supplementary file 1 [file jcpe0040-0349-SD1.docx]

**Figure S2**


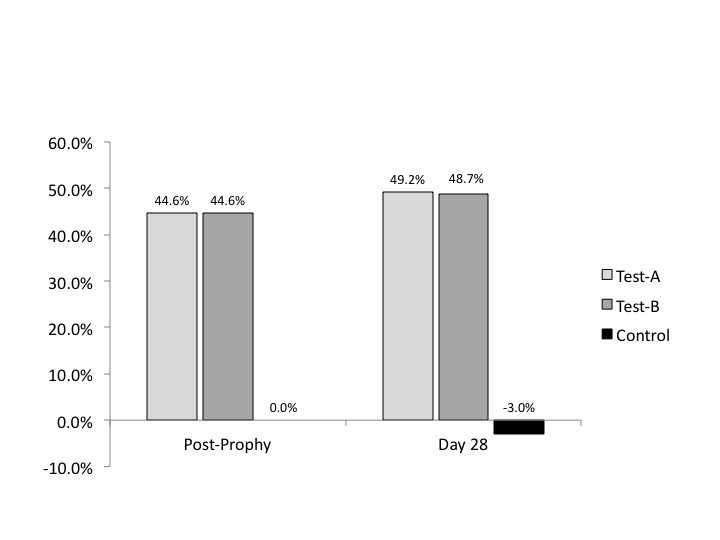

Supplement: Supplementary file 2 [file jcpe0040-0349-SD2.docx]
